# Health sector responses to intimate partner violence: A literature review

**DOI:** 10.4102/phcfm.v6i1.712

**Published:** 2014-11-21

**Authors:** Kate Rees, Virginia Zweigenthal, Kate Joyner

**Affiliations:** 1 Faculty of Health Sciences, School of Public Health and Family Medicine, University of Cape Town, South Africa; 2 Division of Nursing, Faculty of Medicine and Health Sciences, Stellenbosch University, South Africa

## Abstract

**Background:**

Intimate partner violence (IPV) is a common and serious public health concern, particularly in South Africa, but it is not well managed in primary care.

**Aim:**

This review aims to summarise the current state of knowledge regarding health sector-based interventions for IPV, their integration into health systems and services and the perspectives of service users and healthcare workers on IPV care, focusing on the South African context.

**Method:**

PubMed, CINAHL, PsycINFO and Google Scholar were searched between January 2012 and May 2014. All types of study design were included, critically appraised and summarised.

**Results:**

Exposure to IPV leads to wide-ranging and serious health effects. There is sufficient evidence that intervening in IPV in primary care can improve outcomes. Women who have experienced IPV have described an appropriate response by healthcare providers to be non-judgmental, understanding and empathetic. IPV interventions that are complex, comprehensive and utilise systems-wide approaches have been most effective, but system- and society-level barriers hamper implementation. Gender inequities should not be overlooked when responding to IPV.

**Conclusion:**

Further evaluations of health sector responses to IPV are needed, in order to assist health services to determine the most appropriate models of care, how these can be integrated into current systems and how they can be supported in managing IPV. The need for this research should not prevent health services and healthcare providers from implementing IPV care, but rather should guide the development of rigorous contextually-appropriate evaluations.

## Abstrait

**Réponses du secteur de la santé à la violence conjugale: Une analyse documentaire**.

**Contexte:**

La violence conjugale (IPV) est une grave préoccupation commune de santé publique, particulièrement en Afrique du Sud, mais n'est pas bien gérée au niveau des soins primaires.

**Objectif:**

Ce rapport vise à résumer l’état actuel des connaissances en ce qui concerne les interventions sanitaires contre l'IVP, leur intégration dans le système et les services de santé, et les perspectives des utilisateurs des services et des professionnels de la santé sur les soins pour l'IPV, en mettant l'accent sur le contexte sud-africain.

**Méthode:**

On a fait des recherches en 2012 et 2013 sur PubMed, CINAHL, PsycINFO et Google Scholar. On a inclus tous les types d’études qu'on a évalués et résumés.

**Résultats:**

L'exposition à l'IPV entraine toute une série de graves problèmes de santé. Il y a suffisamment de preuves que l'intervention dans le contexte des soins de santé primaires peut améliorer les résultats. Les femmes ayant été victimes d'IPV ont déclaré qu'une réponse appropriée par les prestataires de soins ne portait pas de jugement et était complète et compréhensive. Les interventions d'IPV qui sont complexes, complètes et utilisent des démarches à l’échelle des systèmes ont été très efficaces, mais les barrières au niveau du système et de la société entravent leur mise en œuvre. Il ne faut pas négliger les injustices liées au genre quand on réagit à l'IPV.

**Conclusion:**

D'autres évaluations de la réaction du secteur de la santé à l'IPV seront nécessaires, pour permettre aux services de santé de déterminer les modes de soins les plus appropriés, comment les intégrer dans les systèmes actuels, et comment on peut les aider à gérer l'IPV. La nécessité de mener cette étude ne doit pas empêcher les services de santé et les prestataires de soins de mettre en œuvre les soins pour l'IPV, mais devrait plutôt guider l’élaboration d’évaluations rigoureuses et appropriées dans ce contexte.

## Introduction

Intimate partner violence (IPV) refers to ‘behaviour by an intimate partner that causes physical, sexual or psychological harm, including acts of physical aggression, sexual coercion, psychological abuse and controlling behaviours’.^1^ The World Health Organization (WHO) estimates that 30% of women globally who have been in a relationship have experienced physical or sexual IPV.^2^ In the WHO Africa region, this estimate is as high as 36.6% (95% CI 32.7; 40.5%).^2^ These figures do not include emotional violence which is often omitted from prevalence studies, although it appears to be common and has serious mental health implications.^3^

In South Africa, interpersonal violence is the second-highest contributor to years of life lost^4^ and, in women, IPV accounts for 62.4% of this high burden.^4^ A survey of women in three South African provinces found lifetime levels of physical abuse of between 19% and 28%^5^ and, in Cape Town, 42.3% of working men interviewed reported perpetrating physical violence in a relationship in the previous 10 years.^6^

When emotional abuse is measured, the figures are even higher. A survey conducted in Gauteng found that 43.7% of women reported emotional violence, whilst 65.2% of men reported perpetrating it. Emotional violence was experienced more often than any other type of violence.^7^

Despite this notable burden of disease, standardised protocols for IPV care have not been implemented in the South African primary healthcare services, leading to poor identification and inconsistent management.

This review aimed to summarise the current state of knowledge regarding health sector-based interventions for IPV, their integration into health systems and the perspectives of service users and healthcare workers on IPV care, focusing on the South African context.

## Methods

Multiple searches of PubMed, CINAHL, PsycINFO and Google Scholar were conducted between January 2012 and May 2014. All types of study design were included. Keywords included intimate partner violence; domestic abuse; violence against women; gender-based violence; South Africa; developing countries; intervention; and health systems. The review was undertaken as part of a larger research project whose protocol was approved by the University of Cape Town's Faculty of Health Sciences’ Human Research Ethics Committee (reference 655/2012).

## Review findings

### Health effects

For women experiencing IPV, the negative effects span all aspects of health and can lead to mortality, morbidity and increased risk factors for poor health outcomes. These effects are mediated through multiple pathways, including physical trauma, psychological trauma and stress, as well as controlling behaviours leading to limited reproductive control and lack of autonomy in healthcare seeking.^2^

Mortality can be caused through homicide, or indirectly through suicide,^8^ maternal causes^9^ and an association with HIV.^10^ Increased morbidity results from increased mental disorders, injuries, increased chronic conditions and physical complaints and reproductive health problems, including HIV and other sexually-transmitted infections.^2, 8, 11^

Mental disorders that are most prevalent amongst women who have experienced IPV include depression, post-traumatic stress disorder, suicidal tendencies and alcohol and substance abuse.^12^ A recent study in South Africa found that of women who obtained protection orders against intimate partners, 66.4% experienced severe depression symptoms and 51.9% experienced symptoms of severe post-traumatic stress disorder.^13^ A recent systematic review of longitudinal studies was able to conclude that IPV is associated with incident depression symptoms, adding to evidence of a causal relationship.^14^

Women who have experienced IPV are more likely to report poor overall health and more likely to suffer physical symptoms, including pain.^8^ They are also more likely to have gastrointestinal symptoms and diagnosed functional gastrointestinal disorders, gynaecological disorders and many more physical disorders.^11^

IPV is associated with an increased risk of being HIV positive, even after adjusting for risk-taking behaviours.^10^ Besides the biological risk resulting from forced sex, male perpetrators are more likely to engage in risky behaviours outside of the relationship and are therefore more likely to transmit HIV.^15^ Women who are in controlling or abusive relationships are also less able to negotiate condom use.^2^ Risky behaviours associated with IPV include multiple partners, transactional sex and substance abuse.^10^

Pregnancy outcomes are worse for women experiencing IPV, in terms of both maternal and foetal health. Poor maternal health outcomes include increased rates of sexually-transmitted infections, vaginal bleeding and premature rupture of membranes. Poor foetal outcomes include low birth weight and preterm delivery.^9^ There is also evidence that IPV can be linked to unintended pregnancy.^16^

Women who have a history of IPV have been shown to have significantly higher levels of healthcare utilisation^17^ and the estimated economic burden is significant.^18^ In Canada, the annual cost resulting from IPV amongst women who had recently left a violent relationship was estimated to be 6.9 billion Canadian dollars.^19^ This emphasises that leaving a relationship does not necessarily mean the end of violence or its impacts.

The high global burden of IPV, its numerous health effects, its impact on efforts to prevent HIV and the opportunity afforded to healthcare providers to inquire about violence, have contributed to growing recognition that IPV is an issue of major public health concern.

### Conceptualising intimate partner violence

Many theoretical perspectives and frameworks have been used to explain and guide research on IPV. For example, the feminist perspective highlights patriarchy and male dominance as causes of IPV, whilst the sociological perspective points to prior experiences of violence and unequal resources in relationships.^20^ An ecological framework approach attempts to pull together factors that lead to violence on multiple levels, incorporating individual, relationship, societal and structural causes.^21^ No one theory fully explains IPV; causes are complex, interrelated and contextual with dynamic, non-linear pathways.^22^

A theoretical model developed by Jewkes^22^ describes two community-level factors that operate as necessary causes of IPV. These are gender inequality – or male superiority – and social acceptance of the use of violence to resolve conflict. Both are prevalent features of South African communities.^22^

Poverty interacts with these factors by hampering the ability of women to leave violent relationships; and potentially leading men who are disempowered economically to gain power by exerting dominance over women.^23^ Masculine identity is a possible mediator in the relationship between poverty and IPV, with men living in poverty being unable to fulfil their conceptualisation of masculinity, ultimately leading to violence on their part.^22^

On a relationship level, the existence of conflict, negative styles of conflict-management, as well as alcohol abuse (which also acts on an individual level), are important contributors to IPV. Relationship conflict has been hypothesised to be a mediator in the relationship between both poverty^22^ and alcohol^23^ and IPV, with conflict arising around household finances or one partner's drinking leading to violence. Conflict that is most likely to lead to IPV is related to women contravening accepted gender roles. In South Africa, this often translates to women having multiple partners, women drinking alcohol or conflict about male drinking.^22^

There is evidence that a life course perspective could be useful in understanding IPV risk. Experiences in childhood and early adulthood, such as childhood abuse, earlier age at first sex or forced first sex, have been shown to increase IPV risk in women.^24, 25^ The WHO multi-country study on women's health and domestic violence found that risk was highest when both the woman and her partner experienced a risk factor,^24^ highlighting the importance of prevention approaches that target both men and women.

### The response of the health sector to intimate partner violence

Despite growing recognition that IPV is an important public health issue, there has been a relative lack of evidence regarding the most effective health system responses.^26, 27, 28^ A Cochrane review of IPV interventions concluded that there is insufficient evidence to show whether current health sector-based approaches are effective in reducing violence or improving psychological well-being.^28^ This points to a need for the development of new health sector responses,^29^ as well as more rigorous evaluations of interventions and their integration into health services and systems, particularly in primary care, which is relatively under-represented in the literature.^27, 30^

There is, however, sufficient evidence that intervening for IPV in a primary care setting can be beneficial. A recent systematic review of interventions based in primary care found that 76% of 17 included studies showed an improvement in at least one measured outcome, including reductions in IPV, improvement in health-related quality of life and increased safety-promoting behaviours.^31^ Included studies were largely of United States origin, with only one study originating in South Africa, one in Peru and one in Hong Kong.

The WHO has recently published clinical and policy guidelines for responding to IPV and sexual violence,^1^ synthesising the best available evidence in an attempt to increase the prominence of IPV as a health concern. On a policy level, the guidelines recommend integrating services into existing structures as far as possible, as well as having multiple models of care appropriate for different levels, but prioritising primary care.^1^ These recommendations are all based on very low-quality evidence, reflecting the relative lack of quality evaluations of health system responses. The guideline outlines minimum requirements for an appropriate health sector response, including having clear local policies and protocols, ensuring supportive management including financial resources, providing comprehensive care as well as resource materials, working intersectorally, providing appropriate monitoring and evaluation and providing support for carers.^1^

### Screening and intervening

In primary care, intervention for IPV usually consists of screening or identification of women experiencing IPV, followed either by on-site intervention or referral to further specialised services.^31^ Universal screening for IPV is controversial, although the need to identify cases non-routinely in healthcare settings is widely accepted.^32^ Since 2013, the U.S. Preventive Services Task Force has recommended universal screening for IPV in women of childbearing age.^33^ However, a more recently-published, well-conducted randomised controlled trial (the WEAVE study) found no difference in primary outcomes between women who were screened routinely for violence and a control group.^34^ This trial, in addition to prior evidence,^35, 36^ has led to the expert conclusion that universal screening for IPV is ineffective in improving health.^29^ Although screening is able to identify women experiencing IPV, uptake of interventions is impeded by numerous barriers and is often low and, in the setting of asymptomatic women, current intervention approaches have not been shown to be of benefit.

Inquiring about and discussing violence in specific cases during healthcare encounters (selective screening or case-finding) has been recommended as an alternative approach,^29, 37^ followed by more complex, individualised interventions.^29^ This approach has been demonstrated to be feasible, with a cluster randomised controlled trial showing that training and support can significantly increase the number of women identified and referred to services in the absence of universal screening.^38^

Several trials of IPV interventions in primary care have recently taken place, most of them in developed countries, utilising doctors, nurses and lay providers to deliver interventions either on or off-site.^34, 35, 38, 39^ These interventions commonly use empathic approaches and attempt to empower women by helping them to understand their situation, improve their safety and access community resources.^31^

One randomised controlled trial with two levels of intervention (provision of a referral resource card and a protocol administered by a nurse) found both intervention groups experienced significant decreases in violence, suggesting that even the act of disclosure may be an important driver of change for women experiencing IPV.^39^

### System-level interventions

Reviews of IPV interventions have emphasised that comprehensive, system-wide approaches have been the most -effective.^26, 40, 41^ IPV interventions are complex, requiring more than health care provider training to enable effective programme functioning within a health system. For example, a realist review (focusing on programme mechanisms to understand how and why programmes work) found that providers can be supported by four elements of an IPV programme: institutional support at high levels; effective protocols; -ongoing training; and immediate access to support services.^40^

The evidence base informing the scale-up of IPV interventions and their integration into health systems is lacking.^42^ However, examples of published investigations that do exist provide lessons of interest to those wishing to institute an appropriate response to IPV. In Malaysia, the national scale-up of One Stop Crisis Centres, an integrated health sector response to IPV, was investigated. Factors relating to health system structure and organisation, as well as external policy constraints, were found to be barriers to implementation.^42^ Several system-level factors arising from this case study could be applicable in other contexts. Commitment at policy level was found to be necessary, which could be communicated to service delivery level by incorporating appropriate indicators into routine reporting. Adequate training, as well as adjustments to service delivery, in order to ensure that providers have the necessary time and privacy available to them, were required. Finally, flexibility of the model was important so as to allow for its implementation at different levels of care.

An investigation of the integration of gender-based violence laws into the regional health systems of Spain found institutionalisation to be a challenge.^43^ Advancements were often made through the actions of highly-motivated individuals, raising concerns about sustainability. Budget allocation was found to be a key component of sustaining institutional change. It is also noted that since IPV is complex to respond to, protocols, whilst necessary, were insufficient and need to be supported by adequate training.^43^

In South Africa, *Vezimfilho*, a model health sector response to IPV and HIV, was developed and implemented in four districts.^44^ Important findings from an evaluation of the implementation process included the need for a systemic response, with political commitment, policies, protocols and effective referral systems being essential.^44^ In addition, capacity building needed to include addressing values and attitudes toward IPV and gender norms, as well as interpersonal skills in healthcare providers. Support from managers in the health system and strong relationships between multiple stakeholders were seen as being key to a sustainable approach.^44^ System barriers to implementation included insufficient staff and lack of confidence in managerial support, whilst on a societal level, providers’ attitudes and perceptions relating to gender hampered implementation.^44^ The social barriers relating to gender imply that a comprehensive health sector response requires advocating for wider social change.

This need to engage the wider community to address gender issues has been recognised in recent interventions. A pilot health services-based IPV intervention for pregnant women in Kenya included an explicit focus on community collaboration^45^ embedded within an ecological framework.^46^ Of note is the pilot's innovative use of ‘supported referrals’, where community volunteers provide practical assistance for reaching referral services. This pilot was found to be acceptable and feasible and shows promise in intervening for IPV as well as primary prevention.

### Primary prevention

Primary prevention of IPV, focusing on underlying causes and risk factors, remains a challenge. Two programmes have been tested in South Africa, attempting to address both IPV and HIV by focusing on the gendered nature of these interlinked epidemics.

The Intervention with Microfinance for AIDS and Gender Equity (IMAGE) study implemented a microfinance intervention combined with participatory training that focused on gender and HIV and encouraged community mobilisation. After two years, women enrolled in the intervention group experienced 55% less IPV in the previous 12 months, compared to the control group (risk ratio 0.45, 95% CI 0.23;0.91).^47^ Although it was unable to show a difference in HIV incidence in communities, the trial showed that a structural intervention that empowers households economically can decrease violence.

Stepping Stones is a participatory programme aiming to prevent HIV through improving gender equity in relationships, thereby decreasing sexual risk behaviour. This programme was adapted for the South African context and was both implemented and evaluated through a cluster randomised controlled trial. The trial was unable to show a decrease in HIV incidence, but did demonstrate a decrease in incidence of Herpes simplex virus 2; and men who underwent the intervention reported perpetrating IPV significantly less often after two years of follow-up.^48^

In India, a programme including a process of community mobilisation, aiming to empower the female sex worker community to work together to decrease their risk of HIV, showed a significant reduction in their experiences of violence.^49^ The SASA! model,^50^ currently being trialled in Uganda, uses community mobilisation in an attempt to alter the community-level factors that increase risk for both HIV and violence. In order to do this, community members and community leaders, as well as professionals such as healthcare workers and police officers, are trained and supported in order to engage with their communities, create networks and share key messages that develop as the empowered group is expanded in the community.

Other instances of IPV-prevention programmes exist, for example the SHARE project in Uganda^51^ and a study comparing different combinations of interventions aiming to promote gender equitable behaviour in Brazil.^52^ In addition, a recent systematic review of IPV-prevention interventions specifically targeting adolescents, found that half of the included trials were effective.^53^ The interventions focused largely on building awareness, with some focusing on social norms and gender inequity. Interventions that reported positive outcomes were conducted in multiple settings (e.g. school and community). A trial of a structured home visitation programme for high-risk pregnant women in the Netherlands showed a decrease in IPV in the intervention group.^54^ These examples not only demonstrate that IPV can be prevented through the efforts of the health sector acting in concert with other stakeholders and the community, but also highlight the importance of addressing gender inequity when responding to both IPV and HIV. Approaches that include community empowerment and mobilisation, explicitly recognising that social and structural factors need to be addressed, appear to be particularly successful.

### Women's perspectives on intimate partner violence care

The WHO clinical and policy guidelines for responding to IPV and sexual violence against women advocate for woman-centred care.^1^ Much literature on the topic of women's experiences and expectations of health services in the context of IPV is available. This includes articles synthesising qualitative research in an attempt to increase evidence availability for policy-making and programme design.^55, 56^ There are many points of commonality, bearing in mind that the majority of this research was conducted in developed countries.

Consistently, women who have experienced IPV have described an appropriate response by healthcare providers to be non-judgmental, understanding and empathetic.^55, 56^ Women want their healthcare providers to understand the complexities and consequences of living with violence and the difficulties they face because of it.^56, 57^ They also want confirmation from their providers that what they are experiencing is abuse; and that it is both unacceptable and wrong.^58, 59^

When these features are present, the encounter can be validating and helpful and raising the issue of violence can be viewed as caring.^56^ When they are absent, however, particularly when the healthcare provider neglects the psychosocial aspects of care, the encounter can be detrimental^57, 60^ and can lead to a reluctance to disclose violence in the future.^55^

Another feature women have described as being of central importance is having healthcare providers respect their autonomy.^58^ However, one survey found that 71% of women who disclosed IPV reported feeling that their healthcare provider wanted them to leave their relationship, with 37.5% reporting that they had been advised directly to do so.^61^ Women have also expressed feeling judged when they did not follow the advice of providers to leave their relationship.^56, 59^

Consistent with the need for a non-directive and validating encounter, South African women experiencing IPV reported that counseling was the service they wanted most often (between 36.1% and 45.8% in different provinces).^5^

Common barriers to accessing help through health services include fear of the abuser and fear of having children removed from the home as a consequence of disclosing violence.^55, 56^ Related to the provider, the fear of judgment and not being believed or understood,^56, 57^ as well as the fear of loss of confidentiality, are common barriers.^55, 56^ At a systemic level, the lack of privacy often encountered in healthcare settings, as well as a lack of continuity, can prevent disclosure.^55^

Overall, the appropriateness of the healthcare encounter depends on the empathetic and non-directive attitude of the provider, the attention paid to emotional issues and the maintenance of confidentiality. If these elements are present, having a healthcare provider raise the issue of violence is usually viewed by women experiencing IPV as being both supportive and helpful.^56^

### Healthcare provider perspectives

Access to reproductive health services can be affected significantly by the attitudes of healthcare workers.^62^ In the case of IPV, perceptions regarding the role of the health system and healthcare workers in intervening for IPV, as well as attitudes regarding the underlying causes of violence, can influence how a patient is managed.^63^

Healthcare workers have dual roles, as care givers and community members, often sharing cultural understandings of violence with their communities. They also experience a similar prevalence of violence to the rest of the community.^64^ Ongoing or unresolved experiences of IPV have been found to be an important factor influencing healthcare workers’ responses to violence, impeding their capacity to offer effective care.^65^ The stigma of seeking help for IPV from colleagues may also make it very difficult for healthcare workers to access care. In a rural South African setting, primary healthcare nurses were found to reflect the dominant culture of normalised violence, viewing IPV as intrinsic to relationships and expressing a preference to deal with abuse within the family structure. Both male and female nurses perceived abuse as an often acceptable form of ‘discipline’, often elicited by the actions of abused women. A distinction was drawn between ‘normal’ levels of abuse and abuse resulting in very severe injuries, for which it was considered more acceptable to seek outside help.^64^

Addressing IPV remains a challenge for many healthcare workers, with studies citing numerous barriers to responding appropriately. On a provider level, these include discomfort dealing with emotional issues^66, 67^ and unrealistic expectations about the outcome of intervention.^63^ One survey found that 58% of healthcare workers had unrealistic expectations of IPV interventions.^68^ This highlights the need to address how healthcare workers understand the complexities of violence, in particular the realities of why women remain in violent relationships. The same survey found that providers were able to empathise with women who were financially unable to leave their relationships, but were less empathetic toward middle-class or educated women who did not leave their partners.^68^

In medical culture, the view of the healthcare provider as the decision maker in the patient–provider relationship is prominent.^69^ This can affect how providers see their IPV encounters, in that a woman choosing not to follow advice to either leave the relationship or seek legal redress, could be interpreted as a failure of the interaction. The provider's inability to provide a solution to the problem may be seen as an inability to intervene effectively.^63^

On a systems level, a lack of time has been cited in many different settings.^66, 67, 70, 71^ A Malaysian study found that although providers lacked time within which to deal appropriately with IPV, whether or not this impacted on the provision of care depended on individual providers’ varying interest in responding to violence.^63^

A lack of training is also a common barrier,^71, 72^ with evidence suggesting that those healthcare workers who have had training tend to ask about IPV more often^73^ and intervene more.^74^ A survey of doctors in South Africa reported that only 9.7% of respondents had received any IPV training.^75^ Similarly, a lack of protocols is perceived by providers to inhibit IPV management^66, 72^ and those who have protocols available report assisting patients more often.^74^

Further systemic barriers include ineffective referral networks^67, 71, 72^ and inadequacies of the healthcare setting in terms of creating a trusting and private environment.^66, 70^

There appears to be some disparity between what women experiencing IPV want from health services and what the health system is currently providing. Whilst women feel validated when an understanding of their complex situation is displayed, healthcare providers are undertrained in IPV and may have unrealistic expectations. Whilst women want non-directive counseling and support, healthcare providers may be uncomfortable with psychosocial issues and want to offer assistance in the form of advice, usually to leave the relationship or get legal help. In addition, system-level barriers impact on the ability of providers to offer appropriate care, whilst social and structural barriers impede access. Well-developed intersectoral linkages, with strong support from high-level management, are required to facilitate the provision of IPV care within the health services and in the community.

### Limitations

This was not a systematic review, seeking all available literature in order to assess it methodologically and present valid study findings. This was not its intention, as the review was designed to inform considerations in the design of health sector-based IPV interventions, including the perspectives of women and healthcare providers. Nonetheless, there is the possibility that factors such as the authors’ opinions and omitted studies have influenced the interpretation of the literature.

## Implications and recommendations

Policy makers should ensure that comprehensive policies are in place for responding to IPV in health services and for preventing IPV at a community level. Healthcare providers should be supported in providing appropriate care, taking cognisance of common barriers.

Further evaluations of health sector responses to IPV are needed, both in order to assist health systems to determine the most appropriate models of care and to determine how these can be integrated into current systems in the context of multiple systemic and societal barriers. Further research is needed to explore: how best to support health systems in providing IPV care; how to operationalise intersectoral approaches to IPV in health systems; and how to improve access to, including acceptability of, services.

The need for this research should not prevent health systems from implementing IPV care, but rather should guide the development of rigorous, contextually-appropriate evaluations.

## Conclusion

There is sufficient evidence that IPV is a common and serious public health concern and that addressing IPV in health services has the potential to improve outcomes. Furthermore, in countries such as South Africa, addressing IPV and gender inequality should form part of HIV prevention programmes.

The health sector has a responsibility not only to respond to the underlying violence that women seeking health services experience, but also to work together with other stakeholders and communities to try and address the contextual factors that lead to IPV and HIV. This requires the empowerment of healthcare workers, women and communities, as well as adjustments to system-level barriers that impede care. A comprehensive health sector response to IPV has the potential to significantly improve the health of the population.
